# FPGA-Based Automatic Digital Pole–Zero Cancellation for Nuclear Pulse Spectroscopy Systems: Real-Time Decay Constant Estimation and Adaptive Tuning

**DOI:** 10.3390/s26196078

**Published:** 2026-09-25

**Authors:** Weiqiang Jia, Haiyu Zhao, Jingjia Ke, Kuan Diao, Xiaojun Liu

**Affiliations:** State Key Laboratory of Digital Manufacturing Equipment and Technology, Department of Instrument Science and Technology, School of Mechanical Science and Engineering, Huazhong University of Science and Technology, Wuhan 430074, China; d202380379@hust.edu.cn (W.J.); d202480331@hust.edu.cn (H.Z.); d202670450@hust.edu.cn (J.K.); dk_hust@163.com (K.D.)

**Keywords:** digital pole–zero cancellation, field-programmable gate array (FPGA), digital pulse processing, adaptive signal processing, gamma-ray spectroscopy

## Abstract

In modern radiation sensor systems, the analog output of charge-sensitive preamplifiers (CSA) often suffers from long exponential tails, which severely limit the performance of the sensor system at high counting rates. At high counting rates in nuclear spectroscopy, overlapping exponential pulse tails from charge-sensitive preamplifiers lead to baseline drift and degraded energy resolution. Effective pole–zero cancellation (PZC) is necessary to mitigate these effects; however, conventional PZC tuning is typically manual and repetitive. We present an FPGA-based automatic digital PZC algorithm for nuclear pulse spectroscopy systems that estimates the preamplifier decay time constant online and automatically adjusts the PZC filter in real time. The proposed system combines high-speed digital pulse acquisition with FPGA firmware to achieve robust baseline restoration, pulse deconvolution, and improved energy resolution. Experimental validation with a high-purity germanium (HPGe) detector and a ^60^Co gamma source demonstrates that the automatic PZC achieves near-optimal tail cancellation and recovers accurate pulse amplitudes. Compared to manual tuning, the adaptive approach yields superior baseline stability and approximately 6.8% better energy resolution for the 1332.5 keV peak, while reducing adjustment time from minutes to seconds.

## 1. Introduction

In nuclear pulse spectrometry, the charge-sensitive preamplifier (CSA) produces pulses with a fast leading edge followed by a long exponential tail. At high counting rates, these tails overlap, driving pulse pile-up and baseline drift that bias peak heights and broaden the energy-resolution function; in severe cases, the accumulated charge can also overload subsequent shaping or digitization stages [1,2,3,4,5]. A widely adopted mitigation is PZC, implemented as a modified differentiator that selects resistor–capacitor values to cancel the CSA’s decay component and force a rapid return to baseline without undershoot [6]. In practice, robust performance requires matching the cancellation time constant to the actual CSA decay, which can vary due to detector leakage current, count–rate–induced baseline shifts, and temperature. Mismatch leads to residual undershoot, reintroducing spectral distortion [7,8]. Both analog PZC networks and their digital counterparts (deconvolution-based filters) are used; the latter allow adaptive tracking under varying operating conditions [9,10,11]. Overall, PZC is a critical element of high-rate nuclear pulse-processing chains, enabling higher throughput and improved peak fidelity in Radiation Measurements–type applications [12,13,14,15,16,17,18].

In contemporary digital nuclear pulse measurement systems, the detector signal—after minimal analog conditioning such as gain setting and anti-alias protection—is digitized directly by a high-speed, high-resolution analog-to-digital converter (ADC). Downstream, field-programmable gate arrays or digital signal processors execute pulse detection, amplitude extraction, and timing measurements [19]. This “digitize-early, process-in-firmware” paradigm reduces susceptibility to analog drift and crosstalk while enabling algorithmic flexibility and reproducibility across operating conditions. As a consequence, functions historically realized in the analog domain are now expected to be performed digitally, and digital PZC becomes a foundational step of nuclear pulse filtering and shaping.

Recent years have seen growing interest in adaptive and FPGA-oriented digital pulse processing for nuclear spectroscopy. Representative efforts include alternative shaping families such as trigonometric-function-based Gaussian-like shaping [20] and FPGA-based trapezoidal shaping [21], as well as adaptive schemes that track system variations in real time, e.g., transfer-function tracking with xLSTM [22] and FFT-based adaptive trapezoidal shaping [23]; a comprehensive review of these trends is given in [24]. These works, however, mainly optimize the shaping stage and generally assume that the preamplifier decay has already been properly compensated. Automatic estimation and cancellation of the preamplifier pole—particularly under resource-constrained FPGA implementations—therefore remains an open issue.

Prior work has established the feasibility and benefits of digital PZC. Zhang Huai-qiang and co-workers [25] derived recursive forms for PZC, implemented them for both simulated and measured nuclear pulses, and analyzed frequency-domain filtering characteristics. Related studies further compare parameter choices on synthetic and real data and emphasize that the effectiveness of PZC depends critically on selecting parameters consistent with the input signal’s decay time constant. However, a persistent gap remains at the system-integration level: parameter selection in practice is often manual. Technicians typically enter initial PZC parameters, acquire raw and processed waveforms, inspect tailing and baseline behavior, and iterate until acceptable peak shapes are obtained. This trial-and-error workflow is time-consuming, operator-dependent, and difficult to reproduce; it also struggles to track variations arising from temperature drift, detector aging, or changes in count rate.

This work is of a technical nature and focuses on the engineering development of an adaptive signal-processing solution for nuclear pulse spectroscopy systems. It bridges the gap between analog sensor front-ends and digital smart sensor systems. By embedding the automatic PZC algorithm into the FPGA-host co-design system, we transform the traditional passive radiation detector into an adaptive, intelligent sensor node that can self-calibrate its signal conditioning path according to the real-time operating conditions. To address these limitations, this work derives a criterion for computing optimal digital PZC parameters directly from the observed preamplifier output, and proposes a practical algorithm for digital automatic pole–zero cancellation suitable for real-time deployment. The method estimates the relevant decay behavior from the trailing portion of pulses, translates it into PZC settings without human intervention, and updates those settings as operating conditions evolve. We implement the algorithm in the FPGA-host co-design system and verify its effectiveness using both simulated waveforms and experimental data. For experimental validation, a Canberra GC3018 coaxial HPGe detector was selected. This detector is specifically designed for high-resolution gamma-ray spectroscopy in the medium-to-high energy range (approximately 50 keV to 10 MeV) and is routinely characterized using ^60^Co sources (1173.2 keV and 1332.5 keV), making it a standard and appropriate testbed for evaluating the performance of digital pulse processing algorithms in high-resolution spectroscopy applications. While scintillation detectors such as CsI:Tl or BGO are also used with ^60^Co sources, the superior energy resolution of HPGe detectors makes them far more sensitive to baseline drift and residual tailing, thus providing a more stringent validation platform for the proposed automatic PZC method. The experimental results demonstrate that automatic digital PZC can deliver stable baseline recovery, reduced tailing, and improved spectral fidelity while preserving the compact resource footprint required by embedded spectrometers.

## 2. Principles and Digital Modeling of Nuclear Pulse Pole-Zero Cancellation

The nuclear pulse PZC circuit is shown in Figure 1. It consists of a CSA followed by a PZC stage. As illustrated, the detector output $Q_{i}$ is first applied to the CSA, which integrates the detector charge and produces a fast-rising pulse with a slow return to baseline. The CSA output $V_{1}$ is then routed to the PZC stage, which shapes the waveform to obtain the processed output $V_{2}$. When properly adjusted, the PZC shortens the effective pulse width, accelerates baseline recovery without over- or undershoot, suppresses low-frequency components, and reduces the likelihood of pulse pile-up at high counting rates.

In the CSA stage, $C_{1}$ serves as the integrating capacitor and $R_{1}$ as the discharge resistor. Charge accumulates on $C_{1}$ and then leaks through $R_{1}$, producing at the output $V_{1}$ a fast-rising pulse with a long decay tail. Because $R_{1}$ is typically large, the tail is slow to return to baseline, which increases pulse pile-up and baseline wander at high counting rates. A first-order CR shaper composed of $C_{2}$ and $R_{2}$ is often inserted to shorten the pulse width; however, a plain CR stage introduces undershoot (a negative lobe) that can overload downstream amplifiers or digitizers and destabilize the baseline. The PZC circuit improves upon this by adding an appropriate resistor $R_{p}$ to the CR network so that the dominant pole of the front stage is cancelled by a corresponding zero in the following stage. With proper tuning, undershoot is eliminated while the pulse is still narrowed, yielding faster baseline recovery, reduced low-frequency content, and a lower probability of pile-up. In practice, this produces a cleaner, more stable waveform at the PZC output $V_{2}$ and supports more accurate peak-amplitude extraction; conversely, any mismatch between the CSA decay and the PZC setting leaves residual tailing, so periodic—or automatic—adjustment is advisable.

The CSA circuit, regardless of its specific implementation, typically consists of a feedback resistor $R_{1}$ and a feedback capacitor $C_{1}$ in a parallel configuration. The output signal of the preamplifier is typically a single-exponential signal, whose polarity (positive or negative) depends on the specific CSA circuit implementation. For a pulse whose duration is much longer than its rise time, the decay portion can be well approximated as a single exponential. Assuming its pulse amplitude is $\frac{Q}{C_{1}}$, where Q is the total charge collected by the detector, and the time constant is $\tau =R_{1}C_{1}$, the CSA output can be expressed as:

(1) $$V_{1}(t)=\frac{Q}{C_{1}}\exp\left(-\frac{t}{\tau }\right)$$

whose Laplace transform is

(2) $$V_{1}(t)=\frac{Q}{C_{1}}\cdot \frac{1}{s+1/\tau }$$

with a uniform sampling period $T_{s}$, the discrete-time samples follow:

(3) $$\left\{\begin{array}{l} V_{1}[n]=\frac{Q}{C_{1}}k_{0}^{n}u[n]\;\\ k_{0}=\exp\left(-\frac{T_{s}}{\tau }\right) \end{array}\right.$$

where τ is the pulse signal decay time constant; u[n] is the unit step function. The PZC circuit consists of capacitor $C_{2}$, resistor $R_{2}$, and potentiometer $R_{P}$, Defining $\tau_{1}=R_{p}C_{2},\;\;\tau_{2}=(R_{p}∥R_{2})C_{2}$. Its transfer function is the first-order pole–zero compensator

(4) $$H_{1}(s)=\frac{1+s\tau_{1}}{1+s\tau_{2}}$$

Cascading with the CSA gives

(5) $$V_{2}(s)=V_{1}(s)H_{1}(s)=\frac{Q}{C_{1}}\cdot \frac{1}{s+1/\tau }\cdot \frac{1+s\tau_{1}}{1+s\tau_{2}}$$

Choosing $\tau_{1}=\tau_{f}$ cancels the CSA decay pole with the PZC zero, eliminating the long tail and leaving

(6) $$V_{2}(s)=\frac{Q}{C_{1}}\cdot \frac{1}{s+1/\tau_{2}}\;\;\;\;(\tau_{1}=\tau )$$

Equivalently, in the time domain, the PZC dynamics can be written as:

(7) $$V_{2}(t)+\tau_{2}\frac{dV_{2}(t)}{dt}=V_{1}(t)+\tau_{1}\frac{dV_{1}(t)}{dt}$$

Discretizing (7) with backward-Euler differences $\frac{dV(t)}{dt}=\frac{V[n]-V[n-1]}{T_{s}}\;$ (unconditionally stable for first-order systems) yields the recursion

(8) $$\left\{\begin{array}{l} V_{2}[n]=\frac{\left(1+k_{1})V_{1}[n]-k_{1}V_{1}[n-1]+k_{2}V_{2}[n-1\right]}{1+k_{2}}\;\\ k_{1}=\frac{\tau_{1}}{T_{s}},\;k_{2}=\frac{\tau_{2}}{T_{s}},\;n\geq 1 \end{array}\right.$$

This is a numerically robust, fixed-point–friendly difference equation for the digital PZC block. It maps component values to coefficients via $\tau_{1}=R_{p}C_{2}$ and $\tau_{2}=(R_{p}∥R_{2})C_{2}$, so that tuning $R_{p}$ enforces the pole–zero match $\tau_{1}=\tau_{f}$ while $R_{2}$ controls the residual pulse width. A useful special case connects (8) to the common “deconvolution” PZC used in fully digital chains. When $\tau_{1}=\tau_{f}$ and $\tau_{2}\gg T_{s}$; then (8) reduces to

(9) $$\left\{\begin{array}{l} V_{2}[n]\approx V_{1}[n]-V_{1}[n-1]+aV_{2}[n-1]\\ a\approx \frac{k_{2}}{1+k_{2}}\approx \exp\left(-\frac{T_{s}}{\tau_{2}}\right) \end{array}\right.$$

which is the standard IIR form that cancels the CSA tail and shortens the effective pulse while preserving area. Given a measured $\tau_{f}$ select $C_{2}$ from bandwidth/noise constraints, set $R_{p}=\frac{\tau_{f}}{C_{2}}$ to enforce cancellation, and choose $R_{2}=\frac{\tau_{2}}{C_{2}}$ to meet count-rate and noise targets. In digital implementations, coefficients $\left(k_{1},k_{2}\right)$ can be updated on-line to track drift in $\tau_{f}$, ensuring sustained cancellation without undershoot or baseline bias.

Simulate the exponential signal output by the preamplifier circuit, with a pulse amplitude of 100 mV and a time constant of 100μs. Let the ADC sampling rate be 10 MHz, i.e., Δt = 100 ns. The pulses processed by digital pole-zero cancellation under different parameters $\left(k_{1},k_{2}\right)$ are shown in Figure 2.

At a sampling rate of 10 MHz (Δt = 100 ns), a detector signal with time constant τ=100 μs provides roughly 3500 samples within Figure 2 observation window, enabling clear resolution of pulse shape and tail behavior. With $\tau_{2}=25\;\mu s$, the post–pole-zero decay constant is 25 μs; the pulse width becomes narrower while the peak amplitude is preserved. When $\tau_{1}=100\;\mu s$, $\tau_{1}$ matches τ and correct pole–zero compensation is achieved: the tail shows no overshoot and decays smoothly toward zero following the expected exponential trend. For $\tau_{1}=50\;\mu s$, overcompensation occurs ($\tau_{1}<\tau$): the tail does not cross the baseline yet fails to approach zero within a reasonable interval, producing a gradual tail-up. For $\tau_{1}=20\;\mu s$, undercompensation occurs ($\tau_{1}>\tau$): the tail overshoots the baseline and relaxes slowly, indicating incomplete cancellation.

These simulations corroborated the parameter-selection guideline for digital pole–zero cancellation: $\tau_{2}$ sets the effective decay constant and thus controls pulse width and baseline recovery speed, whereas τ_1_ determines the accuracy of the compensation. The optimal condition is $\tau_{1}=\tau$, yielding a monotonic, exponential return to baseline without overshoot; $\tau_{1}>\tau$ produces undercompensation with tail overshoot, and $\tau_{1}<\tau$ produces overcompensation characterized by a rising tail.

## 3. Design and Implementation of the Automatic Digital Pole-Zero Cancellation Algorithm

### 3.1. Algorithm Design for Automatic Pole-Zero Cancellation

Automatic PZC replaces operator-dependent tuning with data-driven estimation and closed-loop updates, enabling reproducible compensation across detectors and operating conditions. Rather than adjusting parameters by visual inspection of tails, the algorithm estimates the relevant time constants directly from the digitized pulses and applies stability-constrained updates to the PZC filter. Figure 3 contrasts the two approaches: manual PZC relies on intermittent, subjective tuning, whereas automatic PZC performs continuous, objective optimization.

As illustrated in Figure 3, manual digital pole–zero tuning begins with selecting a threshold that maximizes the signal-to-noise ratio (SNR), a step that necessarily involves operator judgment. The operator then inspects the pole–zero–corrected waveform and iteratively cycles through tuning, acquisition, inspection, and re-tuning. Although workable, this procedure is labor-intensive, time-consuming, and prone to suboptimal or irreproducible settings. To automate pole–zero cancellation, two elements must be replaced by data-driven procedures:

Automatic threshold adjustment. The goal is to acquire input data with the highest achievable SNR while preserving valid pulse detection. Mimicking the manual strategy, the algorithm initializes from a conservative low (or high) threshold and adapts it monotonically until the transition between “no detections” and “valid detections” is found, thereby identifying an operating point that maximizes SNR subject to detection integrity.

Automatic estimation of optimal PZC parameters. The optimal PZC parameter equals the input signal’s time constant. The task therefore reduces to rapid, accurate estimation of the decay constant from sampled data, after which the corresponding PZC setting is obtained directly. For an ideal CSA output(i.e., a standard exponential decay), on the tail segment, Q, $C_{1}$, and u[n] are constants and can be grouped into a single constant M, facilitating robust parameter extraction from the tail-only samples.

Thus, the discrete tail of an ideal CSA output can be written as:

(10) $$V_{1}[n]=Mk_{0}^{n},\;\;\;n\geq 0$$

which immediately yields the first-order recursion:

(11) $$V_{1}[n]=k_{0}V_{1}[n-1],\;\;\;n\geq 1$$

with $k_{0}=e^{-\frac{T_{s}}{\tau }}$ (where $T_{s}$ is the ADC sampling period and τ is the decay time constant), the time constant follows from adjacent tail samples as:

(12) $$\tau =\frac{T_{s}}{\ln\left(\frac{V_{1}[n-1]}{V_{1}[n]}\right)},\;\;\;n\geq 1$$

For noise-free data, the two-point estimate is accurate; in practical acquisition, however, quantization and noise may render it unstable. To improve robustness, multiple tail samples are averaged. Summing both sides of the recursion over N+1 points gives

(13) $$\left\{\begin{array}{l} \frac{S_{1}}{S_{2}}=k_{0}=e^{-T_{s}/\tau }\\ S_{1}=\sum_{n=1}^{N}V_{1}[n]\\ S_{2}=\sum_{n=0}^{N-1}V_{1}[n]\\ \sum_{n=1}^{N}V_{1}[n]=k_{0}\sum_{n=0}^{N-1}V_{1}[n] \end{array}\right.$$

Solving for the decay constant yields the multi-point estimator

(14) $$\tau =\frac{T_{s}}{\ln\left(\frac{S_{2}}{S_{1}}\right)}$$

This estimate uses N+1 consecutive tail samples. Its precision depends on ADC resolution, the number of samples, and the SNR; higher SNR and larger N+1 reduce variance, provided that the selected samples lie within the single-exponential tail (i.e., free of pile-up and saturation). Under these conditions, the decay constant τ of each pulse can be obtained automatically, after which the optimal pole–zero compensation parameters $k_{1}$ and $k_{2}$ are determined according to the relations established above. This procedure replaces manual tuning with a reproducible, data-driven parameter selection while maintaining compatibility with standard digital processing chains.

### 3.2. System Implementation Based on FPGA and Host Computer

The digital pole–zero cancellation function is realized through the combined operation of a digital multichannel analyzer (MCA) and a host computer control system. The digital MCA is composed of several functional modules, including gain adjustment, DC offset correction, differential signal conversion, ADC, field-programmable gate array (FPGA) processing, and USB data communication. These modules perform front-end signal conditioning, digitization, and preprocessing before transferring data to the host computer for further analysis.

The host computer software integrates modules for data communication, spectrum visualization, parameter configuration, and algorithm execution. Through coordinated operation, it enables real-time spectrum display, parameter tuning, and the implementation of the automatic pole–zero cancellation algorithm. The interaction and data flow between the digital MCA hardware modules and the host software are illustrated schematically in Figure 4, which shows how digitized detector signals are processed, transmitted, and analyzed under unified control.

As shown in Figure 4, the left block corresponds to the analog front end, which performs essential conditioning—gain setting, DC offset correction, and differential conversion—before the analog waveform is digitized into a discrete sample stream. The central block is the FPGA, which executes real-time digital processing, including pole–zero cancellation, channel shaping, amplitude extraction, and core control. The fast channel trigger reliably identifies the signal’s leading edge for event timing and pile-up rejection. A FIFO buffer is embedded in the control logic; combined with the fast trigger, it enables acquisition of pre- and post-trigger segments of both the raw input and the processed outputs, which are then transferred to the host.

The right block is the host software layer, responsible for spectrum visualization, parameter management, and execution of computationally intensive routines. To exploit module strengths, operations requiring deterministic, low-latency response—digital pole–zero cancellation and shaping—are implemented on the FPGA, whereas automatic pole–zero tuning, which involves non-real-time optimization and more complex decision logic, is executed on the host. High-speed USB links the two to form an integrated acquisition and processing system.

A distinctive feature of this system architecture is the functional partitioning enabled by the combined use of FPGA and host-PC software. The FPGA is dedicated to real-time, latency-critical operations—specifically, digital pole–zero cancellation, trapezoidal shaping, and pulse amplitude extraction—which require deterministic response and are implemented with a compact hardware footprint. In contrast, computationally intensive tasks that do not have strict real-time constraints, such as pulse waveform analysis, decay time constant estimation, and automatic parameter optimization, are offloaded to the host software. This division of labor ensures high real-time performance for the core signal-processing pipeline while maintaining low hardware resource consumption on the FPGA, making the system suitable for cost-effective and portable embedded spectrometer platforms. The resource utilization of the FPGA implementation is modest: the algorithm occupies 2126 logic elements, 42 KB of on-chip RAM, 12 dedicated 18 × 18 multipliers, and one PLL, with no external DDR memory required. This resource footprint is well within the capacity of low-cost FPGAs such as the Cyclone-IV-EP4CE10, confirming the feasibility of deploying the proposed method in resource-constrained embedded spectroscopy systems.

In accordance with Equation (8), real-time digital pole–zero cancellation is realized in the FPGA. The required parameters $k_{1}$ and $k_{2}$ are configured by the host and written to FPGA registers by USB real-time communication. The corresponding logic diagram is depicted in Figure 5, illustrating parameter loading, streaming data paths, trigger-synchronized buffering, and the handoff of processed event records to the host.

The implementation of automatic pole–zero cancellation requires coordinated operation between the FPGA and the host computer. Noise effects must be considered in the process, and SNR is improved by applying multi-point averaging to the sampled data. The main control and algorithm execution are performed on the host computer, while the FPGA executes the real-time data acquisition and basic digital processing. Communication between the two units is achieved through a defined command protocol, enabling the host to send control instructions and parameter updates to the FPGA and to receive processed data for further analysis and display. This cooperative framework ensures stable real-time performance while maintaining flexibility for algorithm optimization and system adjustment. As shown in Figure 6, the software workflow implements the automatic pole–zero strategy outlined in Figure 3, comprising two core stages: automatic threshold determination and automatic calculation of optimal PZC parameters.

Automatic threshold determination. To obtain a high-SNR pulse sequence, an iterative, timeout-guided reduction is employed. Starting from the maximum threshold permitted by the instrument, the threshold is decreased stepwise after each timeout (no valid pulse detected within the timeout window) until a valid pulse sequence is acquired. This procedure yields the highest practicable SNR under current operating conditions while ensuring reliable pulse capture.

Automatic calculation of optimal PZC parameters. The acquired sequence undergoes baseline correction to remove DC components. Using the detected pulse peak as a reference, the segment spanning 90% to 10% of the peak amplitude is designated as the valid tail region. The first 90% of this region serves as the initial calculation window, which is then slid backward sample by sample to improve statistical robustness. For each window position, the decay time constant is computed using Equation (14). The resulting set of time-constant estimates is averaged to suppress noise and quantization effects. According to the established conclusion, the optimal PZC parameters $k_{1}$ and $k_{2}$ are then derived from the averaged time constant using the sampling interval Δt. This procedure provides stable parameter estimates suitable for on-the-fly configuration of the FPGA’s real-time PZC stage.

The selection of the 90–10% pulse-tail region is justified as follows. The portion above 90% of the peak amplitude is close to the pulse peak and may still be influenced by the finite rise time of the preamplifier, which deviates from the ideal single-exponential decay model. Conversely, the portion below 10% of the peak amplitude is dominated by noise and baseline fluctuations, which can severely degrade the estimation accuracy. The 90–10% interval thus captures the most reliable single-exponential portion of the decay while excluding regions contaminated by non-exponential behavior or noise.

Simulation results show that increasing the tail-region range yields only marginal improvements: the relative error decreases from 0.049% (85–15%), to 0.042% (90–10%), and further to 0.040% (95–5%). This diminishing return confirms that the estimator is not critically sensitive to the exact choice of boundaries, and the 90–10% region provides a conservative and safe balance suitable for practical applications.

The sliding-window strategy, in which the first 90% of the selected tail region is used as the initial window and then slid sample by sample toward the tail, serves two purposes. First, it generates multiple estimates of the decay constant from the same pulse; averaging these estimates suppresses the effects of quantization noise and random fluctuations. Second, and more importantly, it provides a reliable basis for quantifying the statistical uncertainty of the estimation. The standard deviation obtained from these multiple window positions offers a meaningful measure of the estimator’s precision, which is essential for assessing the confidence of the obtained decay constant in practical applications.

### 3.3. Digital Signal Shaping After Pole–Zero Cancellation Based on FPGA

Pole–zero cancellation is essentially a first-order high-pass filtering operation. After this step, additional digital shaping, such as trapezoidal shaping, is required to extract the pulse amplitude accurately. For typical radiation detectors, the preamplifier output is usually a decaying exponential pulse. The purpose of trapezoidal shaping is to transform this exponential waveform into an equivalent trapezoidal pulse, so that the overall response exhibits a pronounced low-pass characteristic. The rise and fall times of the trapezoidal pulse are equal, and both the slope length and flat-top duration are adjustable. These parameters can be optimized according to the input pulse shape and count rate: a longer shaping time constant is beneficial for noise reduction, whereas a shorter shaping time reduces pulse pile-up. Trapezoidal shaping provides a flexible compromise between these two requirements.

Classical implementations of trapezoidal shaping are often based directly on convolution in the time domain, which leads to complicated expressions and is not well-suited for hardware realization on an FPGA. To obtain a hardware-friendly algorithm, the method is reformulated into a recursive form. As shown in Figure 7, the basic idea is: first, use pole–zero compensation to convert the exponentially decaying signal into a sequence of unit impulses; then convolve this impulse sequence with the trapezoidal weighting function using a difference–integration structure. After pole–zero cancellation, the discrete-time signal s[n] satisfies:

(15) $$\frac{s[n]}{s[n-1]}=k_{0}$$

where $k_{0}=e^{-\frac{T_{s}}{\tau }}$, $T_{s}$ is the ADC sampling period, τ is the decay time constant of the pulse, and n denotes the sample index. On this basis, the exponentially decaying signal can be recursively deconvolved into a unit impulse sequence δ[n] as:

(16) $$\delta [n]=s[n]-k_{0}s[n-1]$$

The discrete unit-impulse response of the trapezoidal weighting function can be written in piecewise form as:

(17) $$\frac{1}{H}y[n]=\left\{\begin{array}{cc} \frac{n}{J}, & 0\leq n<J-1,\\ 1, & J\leq n<J+K-1,\\ \frac{2J+K-n}{J}, & J+K\leq n\leq 2J+K. \end{array}\right.$$

where H is the amplitude of the trapezoid, J is the number of samples on the rising (and falling) edge, and K is the number of samples on the flat top. Integrating (17) yields a piecewise constant function, which can be expressed in terms of unit step functions as:

(18) $$\frac{J}{H}x[n]=u[n]-u[n-J]-u[n-J-K]+u[n-2J-K]$$

Taking the first difference of (18) gives a weighting function equivalent to a sum of delayed unit impulses

(19) $$\frac{J}{H}p[n]=\delta [n]-\delta [n-J]-\delta [n-J-K]+\delta [n-2J-K]$$

where δ[n] denotes the unit impulse. Substituting (16) into (19) unifies pole–zero compensation and convolution with the trapezoidal weighting function within a single recursive framework: (16) produces the unit-impulse sequence from the input, and (18) realizes the difference form of the trapezoidal convolution.

In the original derivation, the trapezoidal output is obtained by two successive integrations: first integrating p[n] to obtain an intermediate variable x[n], then integrating x[n] to obtain y[n]:

(20) $$\left\{\begin{array}{l} x[n]=x[n-1]+p[n]\\ y[n]=y[n-1]+x[n] \end{array}\right.$$

Eliminating the intermediate variable x[n] leads to a second-order recursion

(21) y[n]=2y[n−1]−y[n−2]+p[n]

Combining (16), (19), and (21), the complete time-domain recursive algorithm from the pole–zero–canceled signal s[n] to the trapezoidal response y[n] can be written as:

(22) $$\left\{\begin{array}{l} \delta [n]=s[n]-k_{0}s[n-1]\\ p[n]=\frac{H}{J}\left(\delta [n]-\delta [n-J]-\delta [n-J-K]+\delta [n-2J-K]\right)\\ y[n]=2y[n-1]-y[n-2]+p[n] \end{array}\right.$$

Because the trapezoidal weighting function has been obtained via integration, the amplitude of y[n] is proportional to the actual input pulse amplitude with a factor related to J. To recover an output amplitude directly corresponding to the physical input pulse amplitude, the final shaped output can be written as:

(23) $$V_{\mathrm{out}}[n]=\frac{y[n]}{J}$$

Equations (22) and (23) provide a unified recursive description for pole–zero compensation and trapezoidal shaping. Only additions, subtractions, and multiplications by the constants $k_{0}$ and $\frac{H}{J}$ are required, which makes the algorithm highly suitable for FPGA implementation. By appropriately choosing J and K, a compromise between noise performance and pulse pile-up can be achieved. Furthermore, if J is selected as a power of two, the factor $\frac{H}{J}$ can be realized by simple shift operations instead of general multipliers, which further reduces hardware cost. A typical architecture expands (22) and (23) into a pipelined data path: the front stage implements pole–zero compensation and multi-tap delayed differencing, while the subsequent stages realize the second-order integration, finally producing a digital trapezoidal pulse with the desired rise time, flat-top duration, and fall time.

## 4. Testing of Digital Automatic Pole-Zero Cancellation

### 4.1. Automatic Pole-Zero Calculation for a Single Pulse Sequence

A test bench was established using the previously developed digital multichannel analyzer (MCA) with pole–zero capability together with the host control software. A Canberra GC3018 HPGe detector was employed to measure a ^60^Co source. The detector bias was set to +3200 V, and the ADC sampling rate was 50 MHz (sampling interval Δt = 20 ns). Automatic PZC was then executed.

For the pulse sequence used in the automatic calculation, 8192 samples were acquired with an SNR of 49.97 dB. The maximum sample value was 1890 at index 253; the 90% and 10% levels of the maximum were 1701 and 189, respectively. Using the detected peak as reference, the valid decay interval was selected from index 519 to 6025 (5507 points). The first 90% of this interval (index 519–5474) served as the initial calculation window and then slid backward sample by sample. Using Equation (14), 552 decay-constant estimates were obtained; their mean was τ=50.2422 μs with a standard deviation of 0.1058 μs. According to the established relation, the optimal parameters were:

(24) $$\left\{\begin{array}{c} k_{1}=\frac{\Delta t}{\tau }=\frac{20\;\mathrm{ns}}{50.2422\;\mu s}\approx 3.98\times 10^{-4}\\ k_{2}=pk_{1}\approx 3.98\times 10^{-3} \end{array}\right.$$

The distribution of estimated time constants appears in Figure 8. After applying automatic PZC, the detector and processed waveforms appear in Figure 9. The raw detector signal shows a large time constant and long tail, with baseline drift and a smaller pulse piled up on the tail of a larger one, which causes amplitude distortion. With the derived parameters, the effective decay constant reduces to approximately 5 μs; the pulse width narrows, the two piled-up pulses separate, the smaller-pulse amplitude recovers, and baseline drift decreases markedly.

After pole–zero cancellation, the signal was processed using trapezoidal shaping, with a rise time of 8 μs and a flat-top duration of 4 μs. The time constant of the signal after pole–zero cancellation was 5 μs, which was taken to be the time constant of the trapezoidal shaper as well. Trapezoidal shaping inherently exhibits a low-pass filtering characteristic, thereby suppressing high-frequency noise, while the flat-top region facilitates accurate extraction of the pulse amplitude. The combined use of pole–zero cancellation and trapezoidal shaping thus provides complementary high-pass and low-pass filtering, forming a complete nuclear pulse signal processing chain and significantly improving the overall pulse shaping performance.

The total time for this single automatic PZC operation is 2.24 s. Parameter computation (time-constant estimation and mapping to $k_{1}$ and $k_{2}$ takes less than 1 ms, while the automatic threshold search—with timeout-driven iterations—accounts for most of the duration.

To systematically evaluate the robustness of the decay-constant estimator under varying noise conditions, additional simulations were performed using synthesized exponential decay signals with a nominal time constant of 50 μs and a sampling interval of Δt = 20 ns, consistent with the experimental setup. Gaussian white noise was added to achieve SNR levels of 20 dB, 30 dB, 40 dB, 50 dB, and 60 dB. At each SNR level, the decay time constant was estimated using Equation (14), matching the experimental configuration. The mean estimated value, standard deviation, and relative error for each condition are summarized in Table 1.

As shown in Table 1, the estimator provides accurate and stable estimates for SNR ≥ 40 dB, with relative errors below 0.1%. When SNR ≤ 30 dB, the estimation becomes noticeably degraded, with a relative error beyond 0.3% and a significantly increased standard deviation, indicating the practical SNR threshold for reliable operation. Under typical experimental conditions, where the SNR exceeds 40 dB (e.g., 49.97 dB in the HPGe detector measurement presented above), the estimator achieves a relative error below 0.1%, confirming its robustness for practical applications. These results are consistent with the theoretical expectation that higher SNR reduces the estimation variance, as noted in the derivation of Equation (14).

### 4.2. Comparison with Manual Pole-Zero Debugging

As a comparison, a manual pole–zero adjustment process was carried out using conventional methods. The optimal time constant obtained manually was 50.4 μs, and the total operation time was approximately 5 min. The threshold determination alone took about 30 s, while the most time-consuming step involved repeatedly entering new PZC parameters, acquiring processed waveforms, and observing their shapes under magnification. Each iteration required roughly 20 s, and more than a dozen iterations were typically needed to reach satisfactory results.

Digital shaping and amplitude measurement were performed using the time constants obtained from both automatic and manual pole–zero adjustment. Figure 10 shows the resulting signals for the two sets of pole–zero parameters. It can be seen that, in the case of manual pole–zero adjustment, the slightly overestimated time constant leads to incomplete cancellation of the preamplifier decay, so the subsequent trapezoidal shaper produces a pulse with a tilted peak, higher on the left and lower on the right, which degrades the accuracy of amplitude extraction. In contrast, the automatic pole–zero algorithm yields a more accurate time constant and thus achieves nearly perfect pole–zero cancellation. As a result, the top of the trapezoidal pulse is very flat, enabling precise determination of the pulse amplitude.

To comprehensively evaluate the performance of the proposed automatic PZC method, a comparative experiment was conducted using both manual and automatic pole–zero tuning procedures. For the manual approach, ten trained operators with experience in nuclear spectroscopy systems were each asked to independently perform the manual pole–zero adjustment procedure following the standard trial-and-error workflow described in Section 3. Specifically, each operator manually adjusted the PZC parameters, observed the processed waveforms, and iteratively refined the settings until acceptable tail cancellation and baseline recovery were achieved. For each operator, the final PZC parameters and the total adjustment time were recorded. Using each operator’s manually tuned parameters, an energy spectrum of the ^60^Co source was then acquired with the same measurement duration. For the automatic approach, the proposed algorithm was executed once after each manual tuning session (i.e., ten independent automatic tuning runs), and the corresponding parameters, adjustment time, and energy spectra were recorded under identical experimental conditions.

The results, summarized in Table 2, report the mean values and standard deviations of the ten independent trials for both methods. As shown in Table 2, compared with manual pole–zero cancellation, the automatic method reduces the adjustment time from 347.40 ± 58.18 s to only 2.21 ± 0.17 s, corresponding to approximately 0.64% of the manual process. In addition, the automatic PZC achieves a decay time constant of 50.241 ± 0.002 μs, which shows significantly smaller variance compared to the manual result (50.465 ± 0.508 μs), demonstrating superior repeatability. Regarding energy resolution, the automatic method enhances the FWHM of the 1332.5 keV peak from 1.90 ± 0.06 keV to 1.77 ± 0.02 keV, representing a 6.8% improvement, and improves the 1173.2 keV peak from 1.79 ± 0.06 keV to 1.66 ± 0.01 keV, representing a 7.3% improvement. The substantially reduced standard deviations for both FWHM values in the automatic PZC case further confirm that the proposed method provides not only improved energy resolution but also significantly better measurement repeatability compared to the manual tuning approach.

To quantitatively evaluate baseline stability, the standard deviation of the baseline level was calculated from baseline segments extracted from the acquired waveforms for each measurement. As shown in Table 2, the manual PZC configuration exhibits a baseline standard deviation of 273 ± 38 μV, whereas the automatic PZC configuration yields 244 ± 9 μV. The reduction in baseline standard deviation from 273 μV to 244 μV represents an improvement of approximately 10.6%, with the standard deviation of the baseline measurements also decreasing from 38 μV to 9 μV, indicating that the automatic method provides not only a more stable baseline but also significantly better consistency across independent trials. This improved baseline stability is attributed to the accurate pole–zero cancellation achieved by the automatic algorithm, which effectively eliminates residual tailing and associated baseline wander. The reduced baseline fluctuation also contributes to the improved energy resolution observed in Table 2, as a stable baseline is essential for accurate amplitude extraction, particularly in high-count-rate spectroscopy applications where pulse pile-up and baseline drift are critical concerns.

### 4.3. Dynamic Tracking Performance

To verify the real-time tracking capability of the automatic PZC algorithm under time-varying conditions, a dynamic experiment was performed using a programmable arbitrary waveform generator (AWG) to simulate the output of a charge-sensitive preamplifier. The AWG was configured to generate exponential pulses with precisely controllable decay time constants, which were injected into the digital MCA for real-time processing. The pulse amplitude was set to approximately 100 mV, and the sampling rate of the MCA was 50 MHz (Δt = 20 ns); the SNR was 50 dB, consistent with the experimental setup described in Section 4.1.

The decay time constant of the generated pulse train was programmed to change in a stepwise manner over time: 50 μs (0–60 s), 10 μs (60–120 s), 100 μs (120–180 s), 49 μs (180–240 s), and 50 μs (240–300 s). This sequence was designed to simulate slow and fast drifts that may arise from temperature variations, detector aging, or other environmental factors in practical nuclear spectroscopy systems. The automatic PZC algorithm was executed continuously throughout the entire 300-s experiment, and the estimated decay constant was recorded to track the temporal evolution of the system response.

The steady-state estimation results for each segment are summarized in Table 3. The corresponding relative errors were all no greater than 0.1%. These results demonstrate that the automatic PZC algorithm can reliably track time-varying decay constants with high accuracy, maintaining estimation errors well within the tolerance required for optimal pole–zero cancellation. When the decay time constant changes, the system is able to complete the identification and tracking process within 2.5 s. Considering that the actual parameter variations in practical detector systems typically occur on a time scale far longer than this (e.g., hours to months), the proposed system can be regarded as exhibiting excellent tracking performance for decay-constant variations. In routine operation, a longer periodic tracking interval—such as one hour or one day—can be configured to continuously monitor slow drift in the detector signal and periodically update the decay time constant accordingly.

It should be noted that although the AWG provides an ideal test signal with precisely defined parameters, the tracking performance observed in this controlled experiment is representative of the algorithm’s behavior under real detector conditions, as the same signal-processing chain and estimation procedure are employed. The results confirm that the proposed method is capable of adapting to slow drifts in the preamplifier response, which is essential for sustained optimal energy resolution over extended measurement periods under changing environmental conditions.

Compared with manual pole–zero adjustment, the automatic pole–zero scheme is capable of intelligently sensing changes in detector type and adaptively switching to the corresponding signal characteristics, while also automatically tracking slow drifts in the response of a given detector. Through such automatic sensing, computation, and tracking of the pulse parameters, improved energy resolution and reduced peak-position drift can be achieved. This approach is therefore well suited for deployment in various online or fully automated monitoring systems.

### 4.4. Performance at Different Count Rates

To evaluate the performance of the automatic PZC algorithm under high-count-rate conditions, a series of measurements was performed at varying input count rates. The input count rate was obtained from the fast-channel trigger count in the FPGA, which provides a direct measurement of the true pulse arrival rate from the detector. The count rate was adjusted by changing the distance between the ^60^Co source and the HPGe detector while keeping all other experimental parameters unchanged. For comparison, measurements were also performed without PZC (i.e., the PZC stage was bypassed) under identical conditions. After automatic PZC, the time constant was 5 μs, and 50.24 μs without PZC. The shaping time was set equal to the time constant. At each count-rate setting, the input count rate, throughput (output count rate after pulse processing), pile-up fraction, and FWHM of the 1332.5 keV peak were recorded for both configurations. The results are summarized in Table 4.

As shown in Table 4, the automatic PZC significantly improves the throughput across all count-rate conditions compared to the no-PZC case. At an input count rate of 10 kcps, for example, the throughput increases from 3.679 kcps (no PZC) to 8.867 kcps (automatic PZC), representing a 2.4-fold improvement, while the pile-up fraction is reduced from 63.21% to 11.33%. This substantial improvement is attributed to the effective tail cancellation achieved by the automatic PZC, which shortens the effective pulse width and enables the system to process pulses more rapidly without being limited by long exponential tails.

Regarding energy resolution, the automatic PZC consistently achieves lower FWHM values than the no-PZC configuration across the entire count-rate range. At 1 kcps, the FWHM improves from 1.94 keV to 1.75 keV, and at 50 kcps, it improves from 2.85 keV to 1.87 keV, demonstrating the superior baseline recovery and tail cancellation provided by the automatic PZC algorithm. It is notable that as the input count rate increases beyond 20 kcps, the throughput of the no-PZC configuration actually decreases due to severe pulse pile-up, while the automatic PZC continues to maintain increasing throughput up to 50 kcps, confirming the robustness of the proposed method under high-count-rate conditions. These results confirm that the proposed automatic PZC algorithm is more suited for high-count-rate nuclear spectroscopy applications, providing substantially higher throughput, reduced pile-up, and improved energy resolution across all tested count-rate conditions compared with no-PZC condition.

## 5. Conclusions

This paper studies the digital implementation of preamplifier and pole-zero cancellation circuits for nuclear pulse signals, derives digital models for these modules, and verifies them through simulation. By comparing the principles of manual and digital pole-zero cancellation, and based on the derived calculation model for the time constant of exponential decay pulse signals, an automatic pole-zero algorithm is proposed. A hardware and software test platform for digital automatic pole-zero cancellation has been built. The algorithm is verified using a high-purity germanium detector, and good results are obtained. The automation of digital pole-zero parameter debugging and the function of digital automatic pole-zero cancellation are successfully realized.

The proposed calculation model for estimating the time constant of exponential decay pulse signals can be widely used for the accurate calculation of nuclear pulse signal time constants. The digital automatic pole-zero algorithm can be directly applied to the realization of automatic pole-zero cancellation in digital multichannel analyzers.

## Figures and Tables

**Figure 1:**
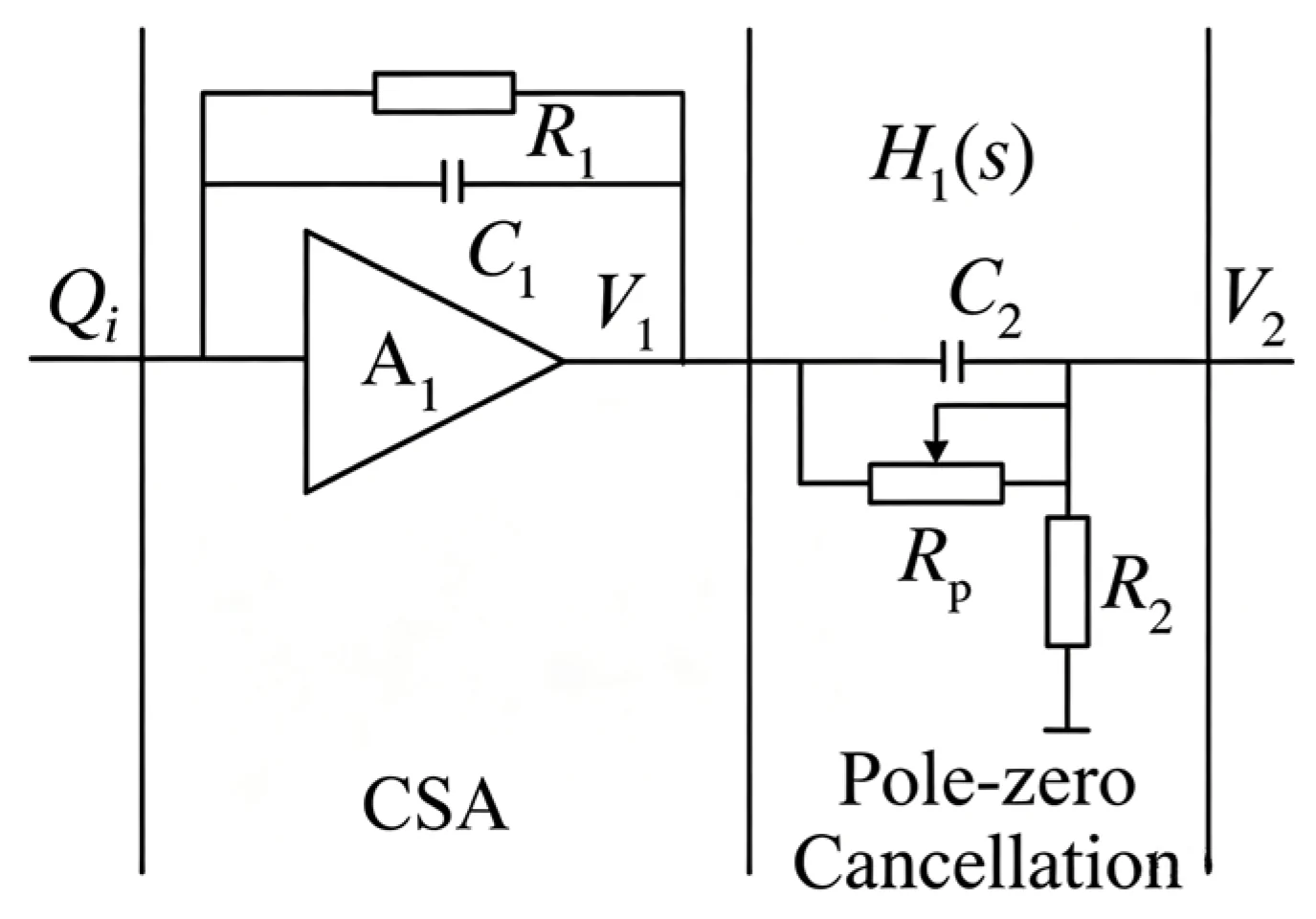
Nuclear pulse signal pole-zero cancellation processing circuit.

**Figure 2:**
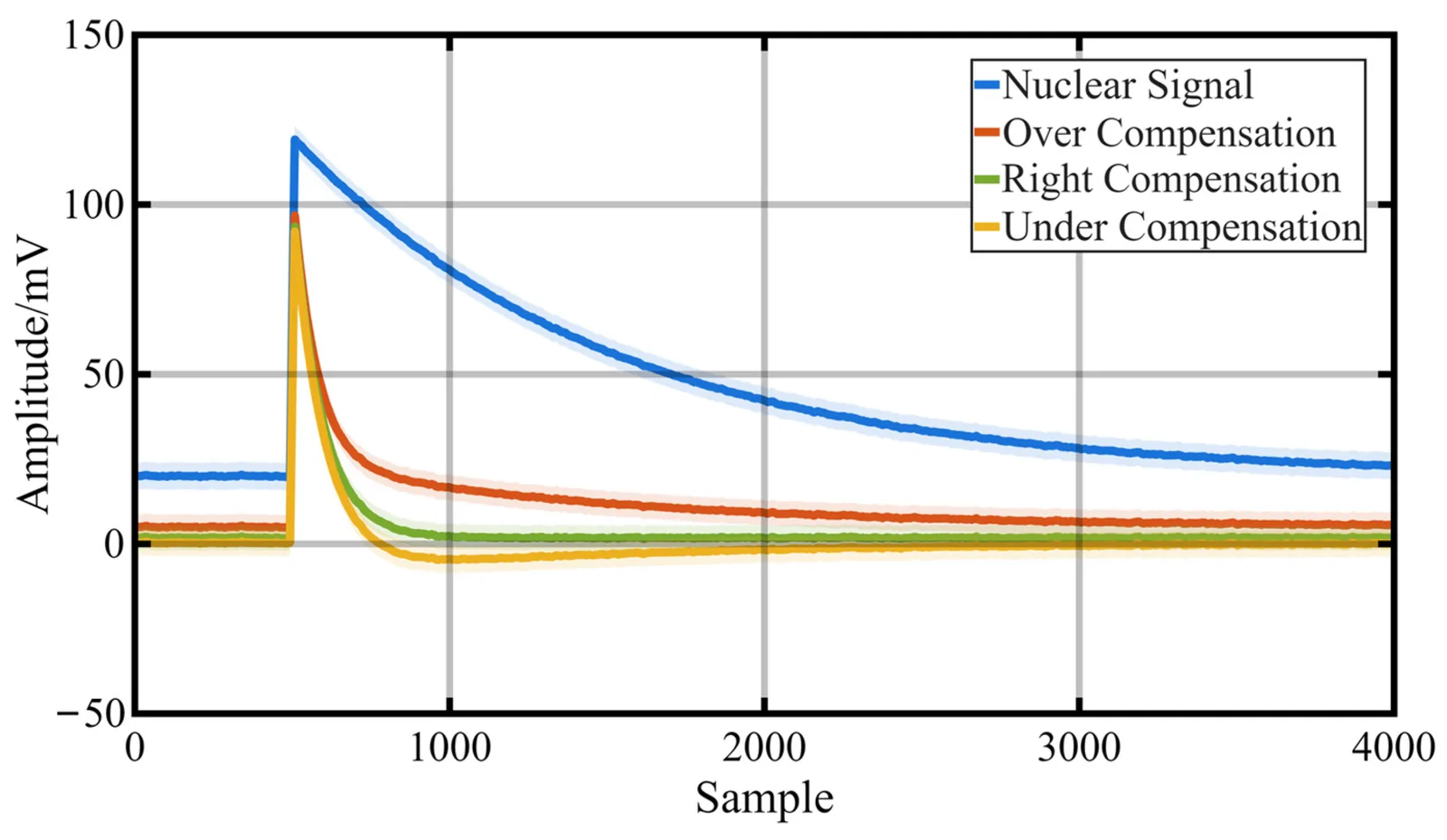
Digital pole-zero cancellation of simulated nuclear signals.

**Figure 3:**
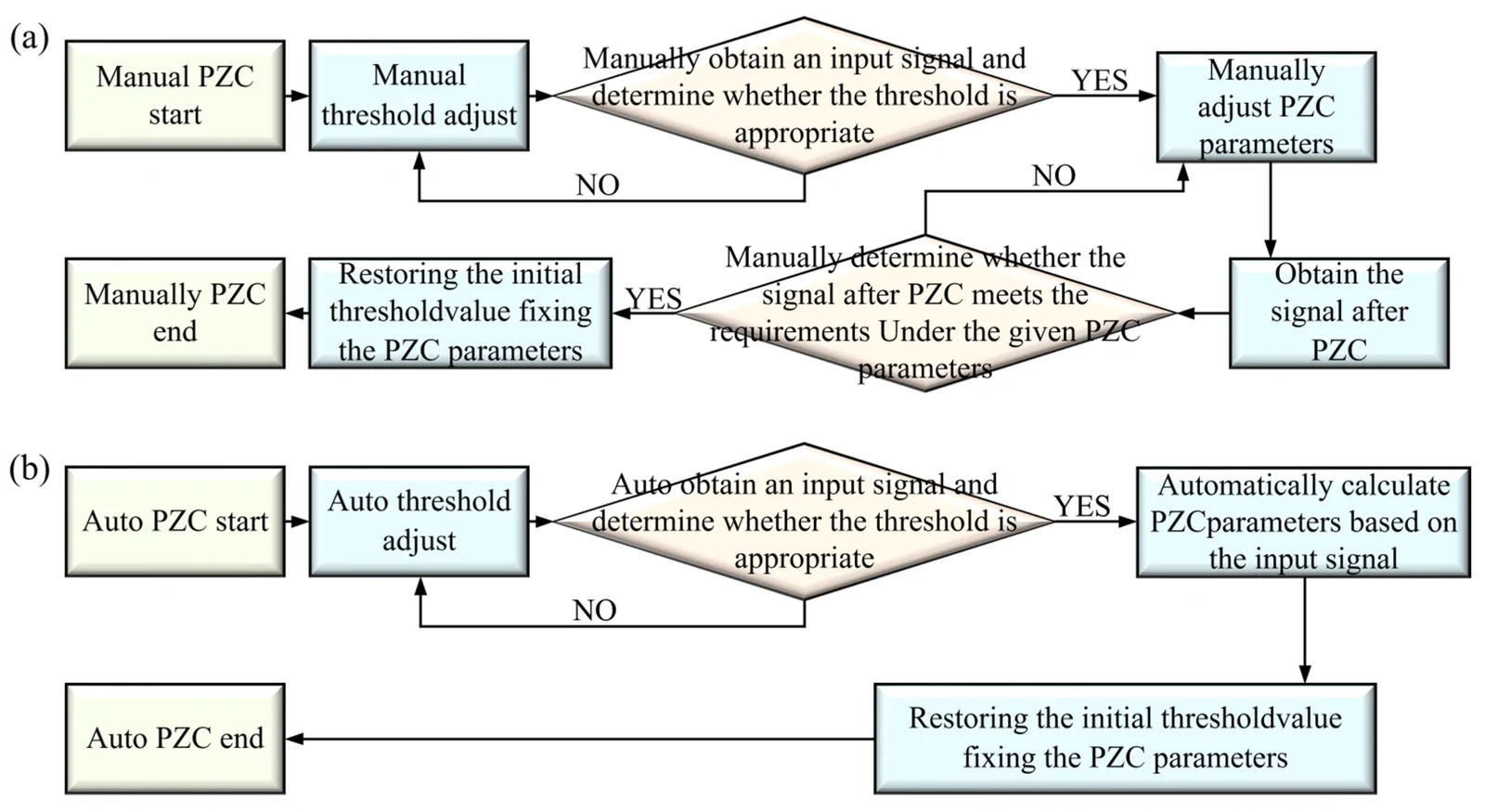
Comparison of manual and automatic pole-zero cancellation principles: (**a**) Manual PZC principle; (**b**) Automatic PZC principle.

**Figure 4:**
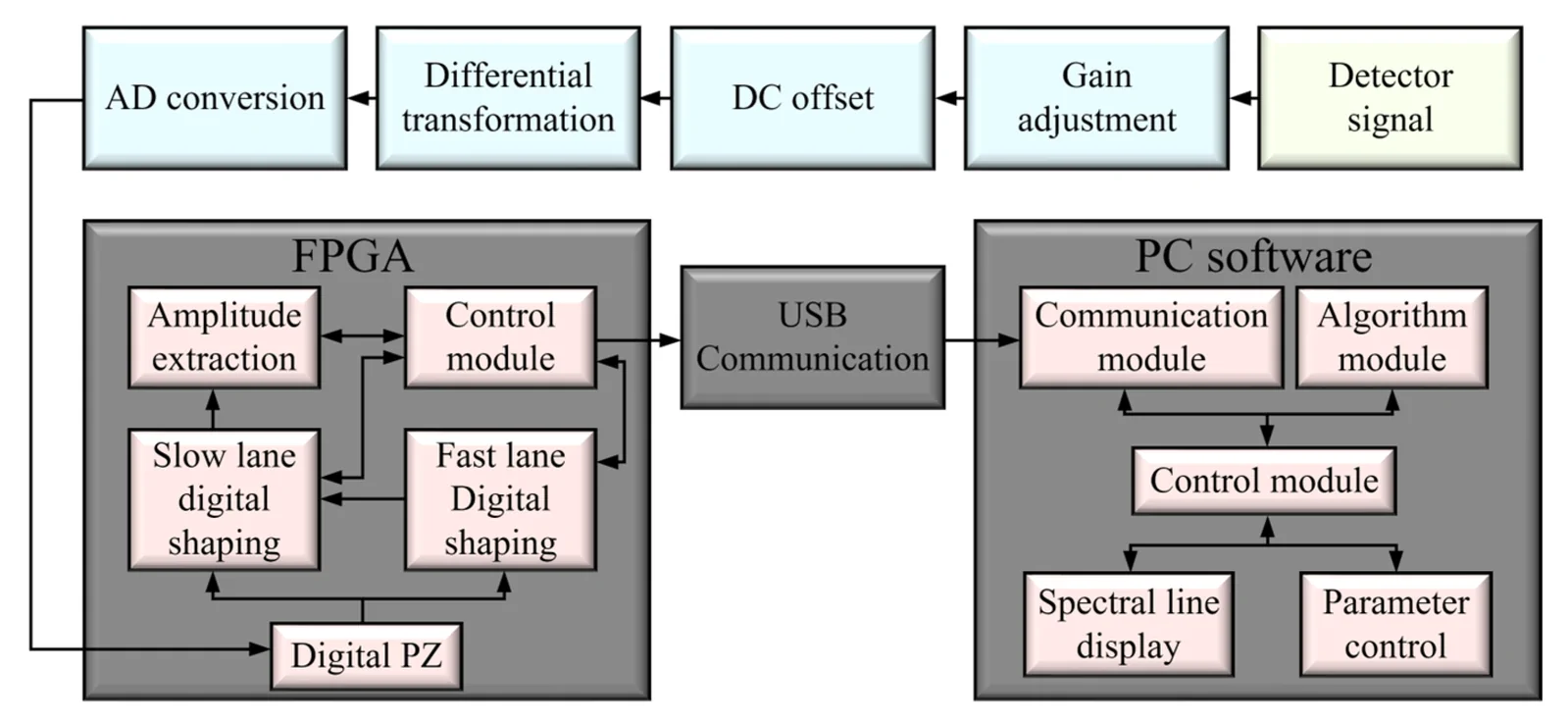
Hardware and software modules of digital automatic pole-zero function.

**Figure 5:**
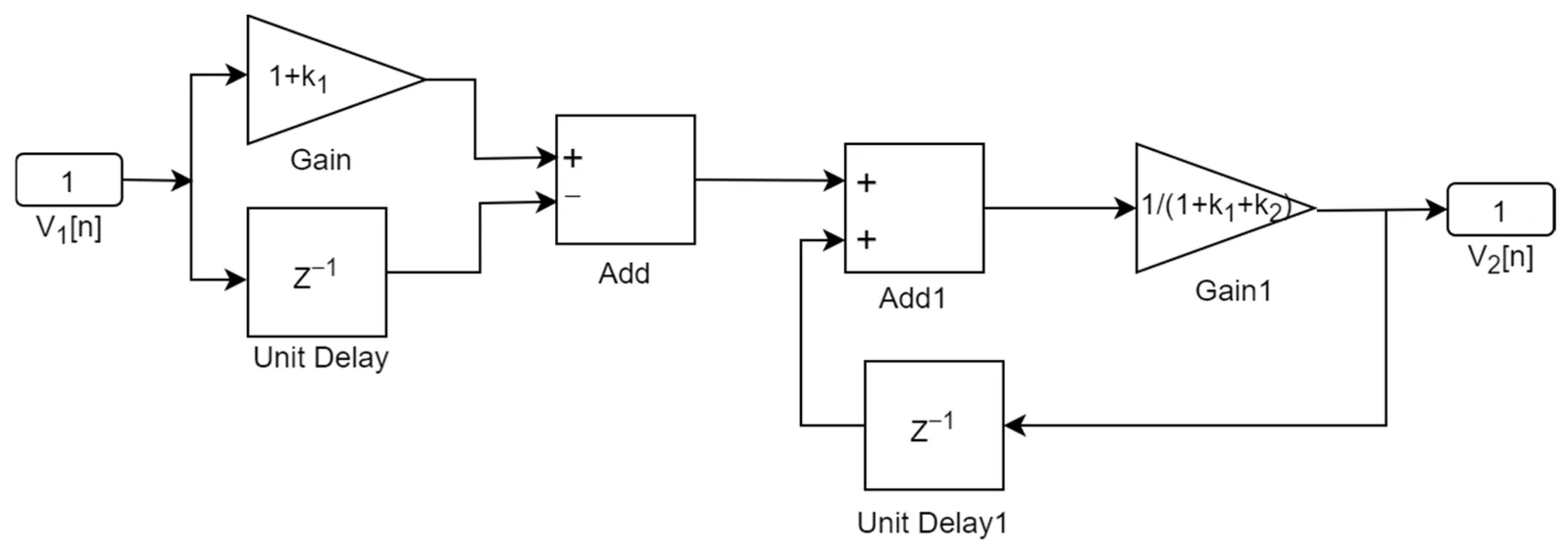
FPGA logic diagram for real-time pole-zero cancellation.

**Figure 6:**
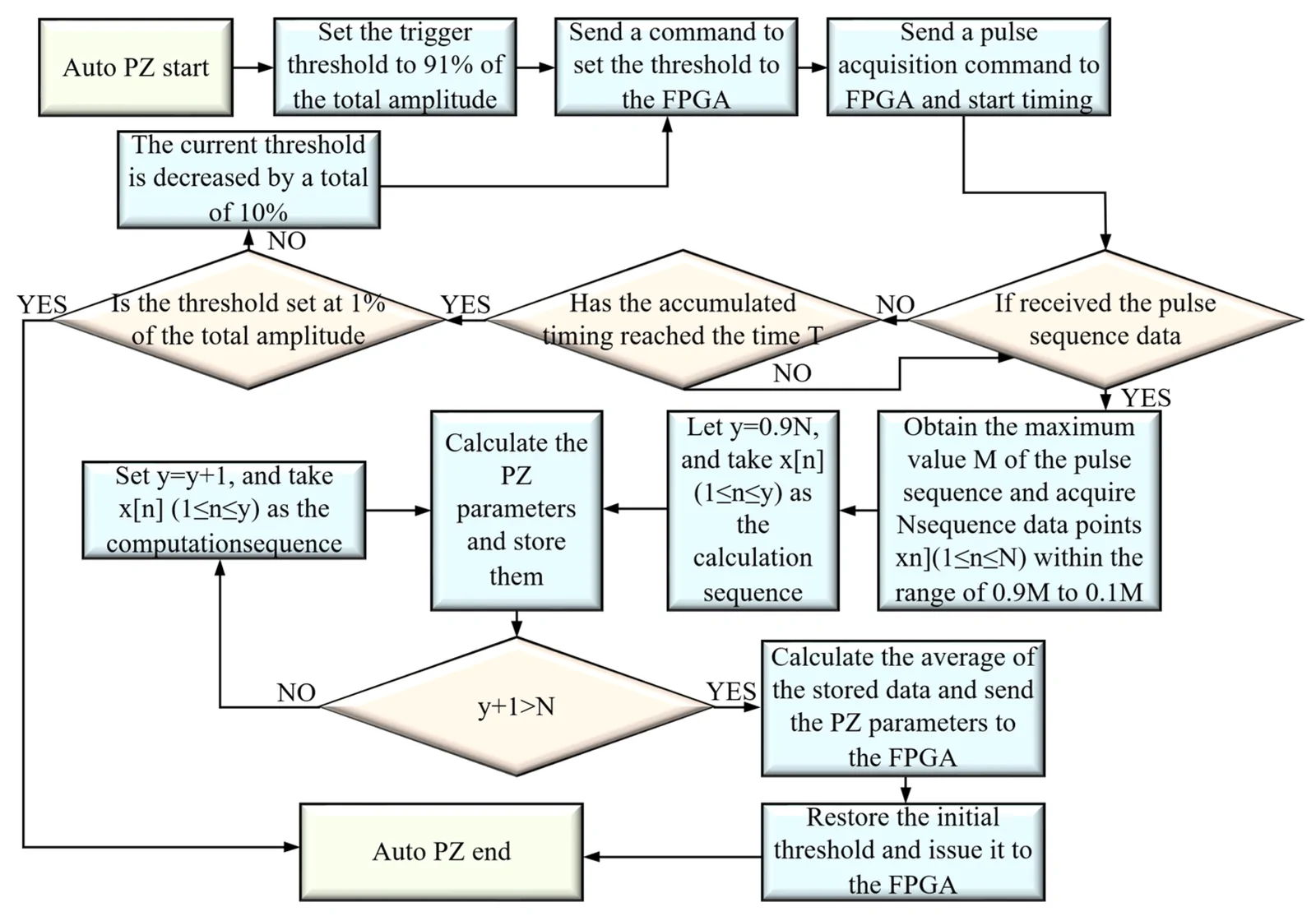
Software flowchart of automatic pole-zero cancellation.

**Figure 7:**
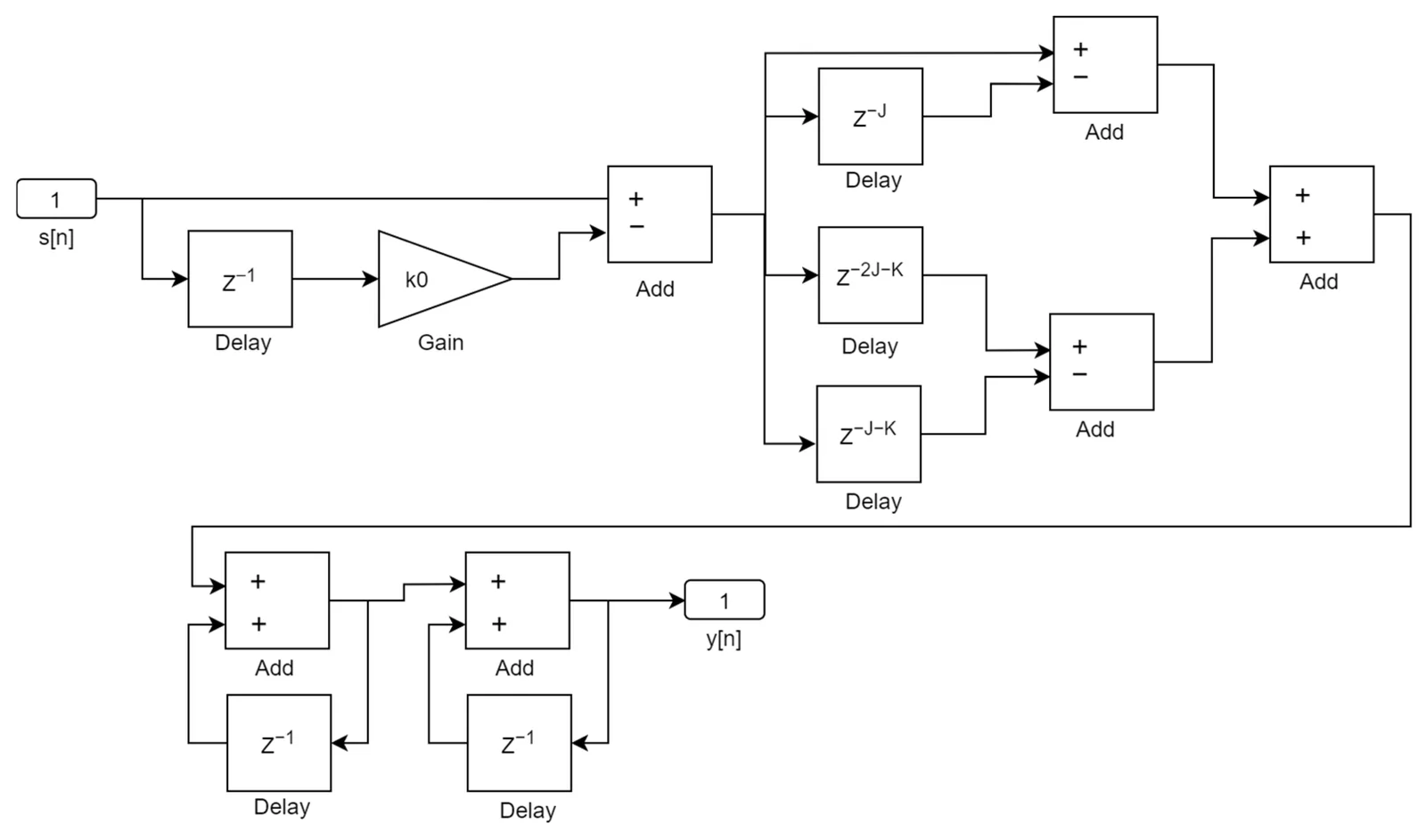
Logic diagram of the digital trapezoidal shaper.

**Figure 8:**
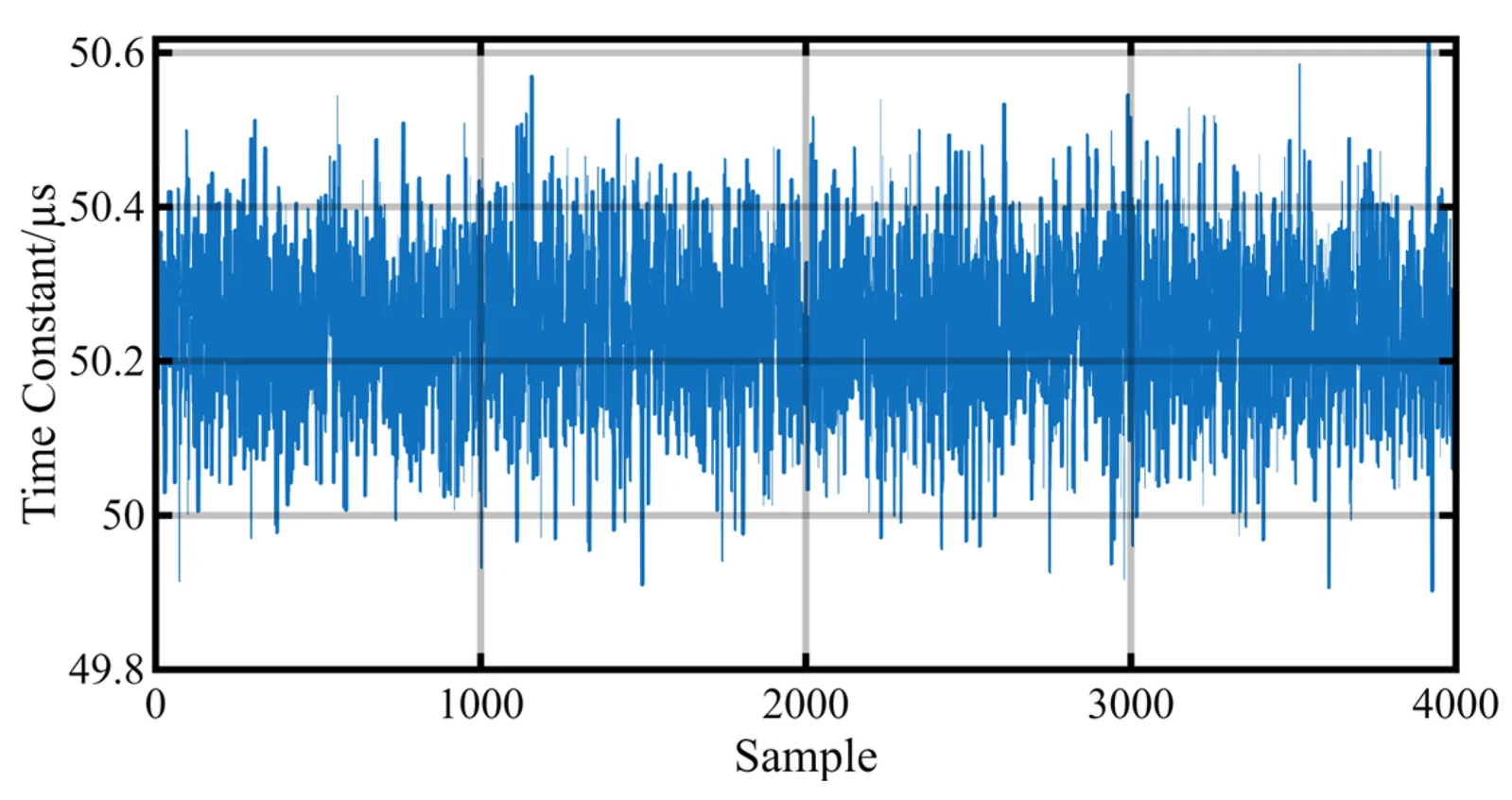
Set of calculated time constant values.

**Figure 9:**
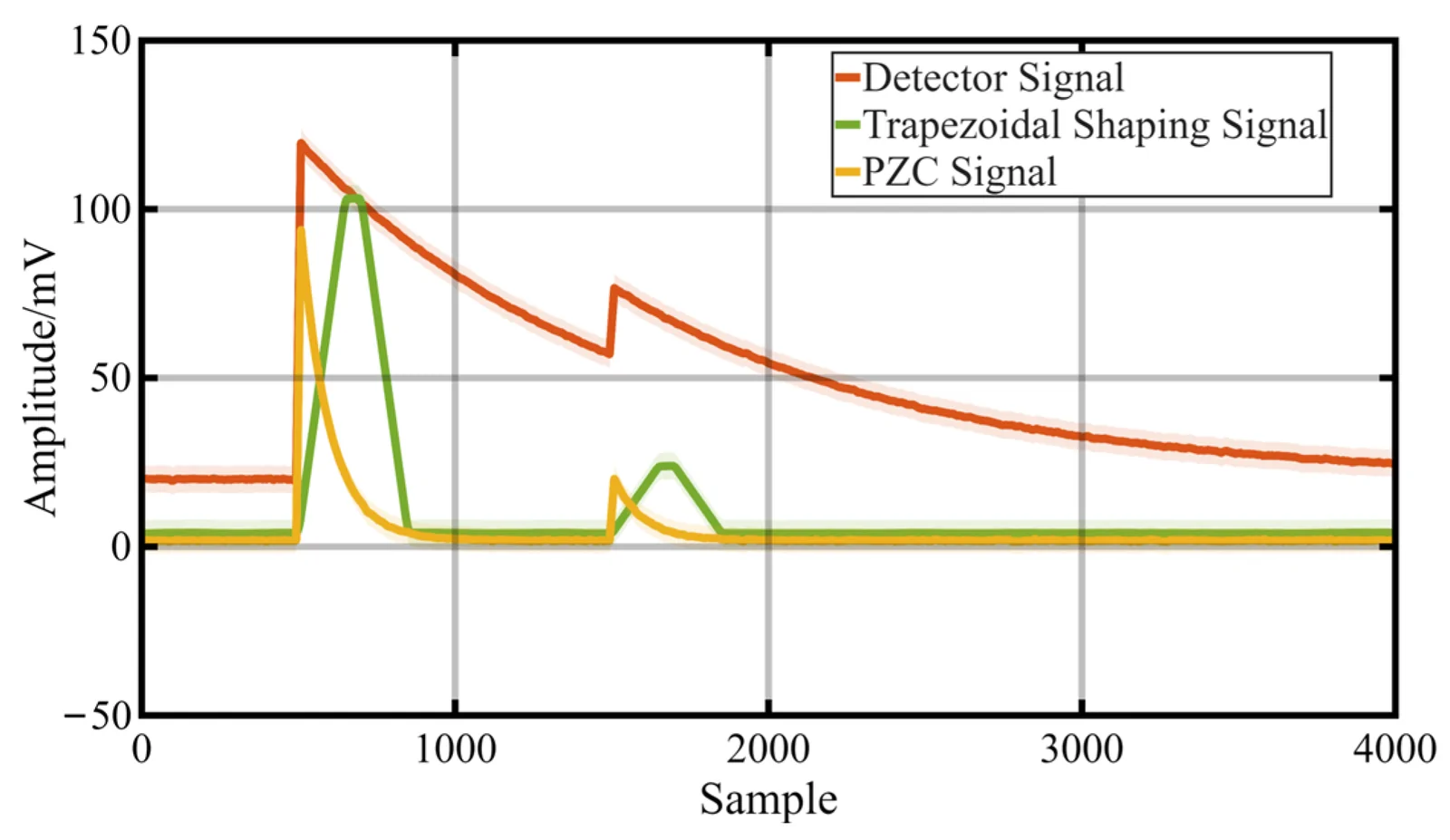
Digital pole-zero cancellation of HPGe detector signal.

**Figure 10:**
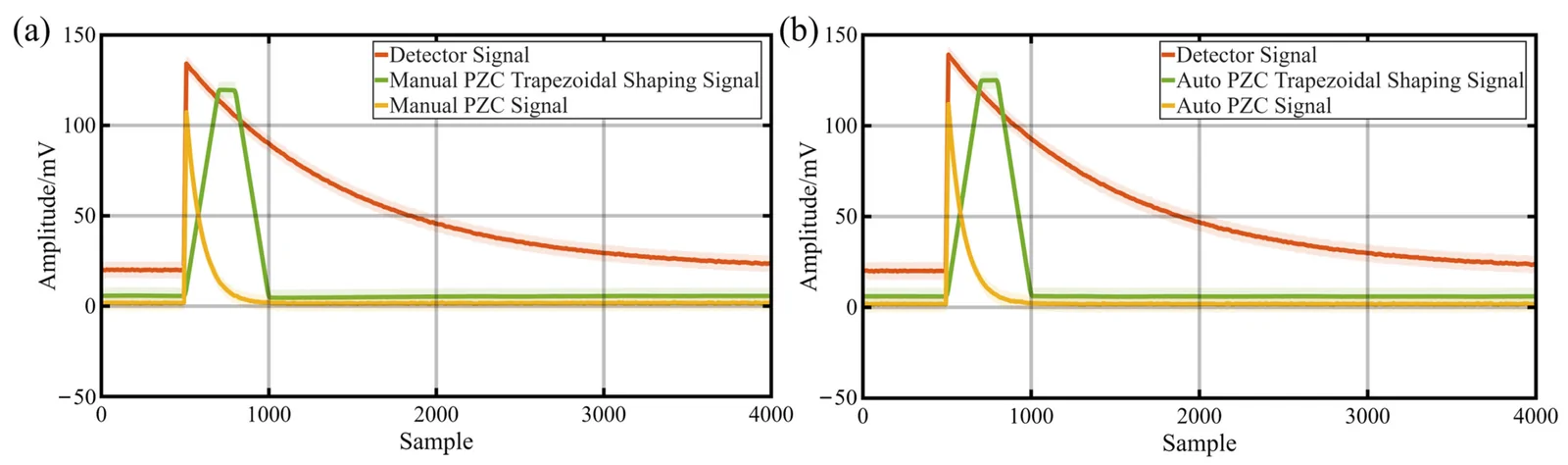
Comparison of trapezoidal shaping results with different pole–zero time constants: (**a**) Manual pole–zero adjustment; (**b**) Automatic pole–zero adjustment.

**Table 1:** Performance of the decay time constant estimator under different SNR levels.

SNR (dB)	Time Constant (μs)	Standard Deviation (μs)	Relative Error (%)
20	52.1145	9.8302	4.229
30	50.1509	2.4242	0.302
40	50.0300	0.7618	0.060
50	50.0221	0.1327	0.044
60	50.0080	0.0531	0.016

**Table 2:** Comparison between manual and automatic pole-zero cancellation.

Method	Time Constant (μs)	Time Used (s)	FWHM @1332.5 keV (keV)	FWHM @1173.2 keV (keV)	Baseline Stability (μV)
Manual PZC	50.465 ± 0.508	347.40 ± 58.18	1.90 ± 0.06	1.79 ± 0.06	273 ± 38
Automatic PZC	50.241 ± 0.002	2.21 ± 0.17	1.77 ± 0.02	1.66 ± 0.01	244 ± 9

**Table 3:** Dynamic tracking performance of the automatic PZC.

Time Interval (s)	True τ (μs)	Estimated τ (μs)	Standard Deviation (μs)	Relative Error (%)	Delay (s)
0–60	50.00	50.02	0.11	0.04	2.15
60–120	10.00	10.01	0.03	0.10	2.04
120–180	100.00	99.97	0.20	0.03	2.24
180–240	49.00	48.98	0.12	0.04	1.99
240–300	50.00	50.01	0.09	0.02	2.11

**Table 4:** Performance comparison between no PZC and automatic PZC at different input count rates.

	NO PZC (τ = 50.24 μs)			Automatic PZC (τ = 5 μs)		
Input Count Rate (kcps)	Throughput (kcps)	Pile-up Fraction (%)	FWHM @ 1332.5 keV (keV)	Throughput (kcps)	Pile-Up Fraction (%)	FWHM @ 1332.5 keV (keV)
1	0.905	9.52	1.94	0.988	1.19	1.75
5	3.033	39.35	2.03	4.709	5.82	1.81
10	3.679	63.21	2.12	8.867	11.33	1.83
20	2.706	86.47	2.37	15.732	21.34	1.83
50	0.335	99.33	2.85	27.440	45.12	1.87

## Data Availability

The raw data supporting the conclusions of this article will be made available by the corresponding author upon reasonable request.
